# Functionalized Graphene Fiber Modified With MOF-Derived Rime-Like Hierarchical Nanozyme for Electrochemical Biosensing of H_2_O_2_ in Cancer Cells

**DOI:** 10.3389/fchem.2022.873187

**Published:** 2022-03-22

**Authors:** Wei Huang, Yun Xu, Yimin Sun

**Affiliations:** ^1^ Hubei Key Laboratory of Plasma Chemistry and Advanced Materials, School of Materials Science and Engineering, Wuhan Institute of Technology, Wuhan, China; ^2^ Key Laboratory of Material Chemistry for Energy Conversion and Storage, Ministry of Education, School of Chemistry and Chemical Engineering, Huazhong University of Science & Technology, Wuhan, China

**Keywords:** graphene fibers, hierarchical structure, flexible microelectrode, nanozymatic electrochemical sensor, *in situ* cancer cell detection

## Abstract

The rational design and construction of high-performance flexible electrochemical sensors based on hierarchical nanostructure functionalized microelectrode systems are of vital importance for sensitive *in situ* and real-time detection of biomolecules released from living cells. Herein, we report a novel and facile strategy to synthesize a new kind of high-performance microelectrode functionalized by dual nanozyme composed of rime-like Cu_2_(OH)_3_NO_3_ wrapped ZnO nanorods assembly [Cu_2_(OH)_3_NO_3_@ZnO], and explore its practical application in electrochemical detection of hydrogen peroxide (H_2_O_2_) released from living cells. Benefiting from the merits of the unique hierarchical nanohybrid structure and high catalytic activities, the resultant Cu_2_(OH)_3_NO_3_@ZnO-modified AGF microelectrode shows remarkable electrochemical sensing performance towards H_2_O_2_ with a low detection limit of 1 μM and a high sensitivity of 272 μA cm^−2^ mM^−1^, as well as good anti-interference capability, long-term stability, and reproducibility. These properties enabled the proposed microelectrode-based electrochemical platform to be applied for *in situ* amperometric tracking of H_2_O_2_ released from different types of human colon cells, thus demonstrating its great prospect as a sensitive cancer cell detection probe for the early diagnosis and management of various cancer diseases.

## Introduction

Since cancer is the second leading cause of death globally, there has always been an increasing research interest in developing efficient techniques for early cancer diagnosis and treatment to circumvent the challenges being faced in clinical practices (Labib et al., 2016; Lin et al., 2016; Asif et al., 2018b). Hydrogen peroxide (H_2_O_2_), one of the most common reactive oxygen species, performs as a critical component within biological systems (Lippert et al., 2011; Aziz et al., 2019a). The level of H_2_O_2_ in human body can be regarded as a versatile biomarker for early diagnosis of various diseases such as cancer (Xiao et al., 2016). For effective and accurate monitoring of the versatile biomarkers associated with cancer, electrochemical biosensors have comprehensive advantages of fast response, low detection limit, high sensitivity, good reproducibility, easy operation, and the ability to be miniaturized for online, and *in vivo* analysis (Sun et al., 2015; Sun et al., 2016; Zhao et al., 2021). Moreover, the development of wearable and implantable electrochemical sensing systems for continuous monitoring of the H_2_O_2_concentration in the human body has shown greater prospects in clinical diagnosis (Wang L. et al., 2018; Asif et al., 2022). Therefore, it is of great significance to construct structural rigidity, high mechanical strength functionalized microelectrodes, which can be integrated into wearable flexible electrochemical sensors to achieve fast, real-time, and high-sensitivity detection (Aziz et al., 2021b).

The development of advanced electrochemical sensors necessitates the design and construction of freestanding electrode system with good mechanical strength and high flexibility (Aziz et al., 2021a), which can be rolled up for use as well as good sensing performance in cancer cell detection. In this regard, graphene fiber (GF), a new type of carbon fiber assembled from graphene nanosheets, possesses one-dimensional microstructure with good flexibility, mechanical strength, high electronic conductivity, and excellent electrochemical stability (Xin et al., 2015; Huang et al., 2018; Xin et al., 2019). The prepared GF integrates the high electrical conductivity of graphene nanosheets and the unique mechanical properties of its macroscopic fiber structure, which has broad application prospects in flexible energy storage/conversion devices, wearable devices, artificial intelligence, etc. (Zhang et al., 2016; Huang et al., 2018). In particular, the intrinsic advantages GF microelectrode, such as adjustable active sites, high signal-to-noise ratio, and good biological compatibility, as well as immobilization of enzyme, enable its application in electrochemical sensing fields (Ding et al., 2016; Chen et al., 2019; Zeng et al., 2019). However, among different kinds of electrochemical sensors, GF microelectrode-based electrochemical biosensor is still in its infancy, so rational design of nanohybrid GF with active components is important for improving the comprehensive performance of electrochemical sensors for the detection of biomolecules. Nanozyme is a class of nanomaterials with enzyme-like characteristics (Wang H. et al., 2018; Wu et al., 2019). With the recent advance of nanotechnology with biology, a variety of nanozymes, such as carbon-based nanomaterials (Ji et al., 2021; Li et al., 2021), transition metal dichalcogenides/peroxides/oxides nanosheets (Asif et al., 2018a; Asif et al., 2019; Huang et al., 2019), noble metal nanoparticles (NPs) (Shen et al., 2015; Xu et al., 2018), and their hybrids (Zhang et al., 2019), have been discovered to possess unique enzyme-mimic catalytic activities and used in the biomedicine or bioanalysis by virtue of their reasonable stability, low cost, mass production, and long-term storage properties that are superior to nature enzymes. In the past few years, tremendous research efforts have been devoted to developing nanozyme-modified GF microelectrodes, which have been used in electrochemical detection of versatile biomarkers, such as adrenaline (Zeng et al., 2019), glucose (Chen et al., 2019), dopamine (Cai et al., 2016; Aziz et al., 2019b), and H_2_O_2_ (Peng et al., 2018; Zhao et al., 2020; Guo et al., 2021). Although considerable progress has been made in this field, it is still a challenge for the development of high-performance functionalized GF-based microelectrodes with optimized microstructural configurations by judiciously selecting electroactive species and rationally designing the composition and structure of nanozyme for continuous detection of biological samples.

**FIGURE 1:**
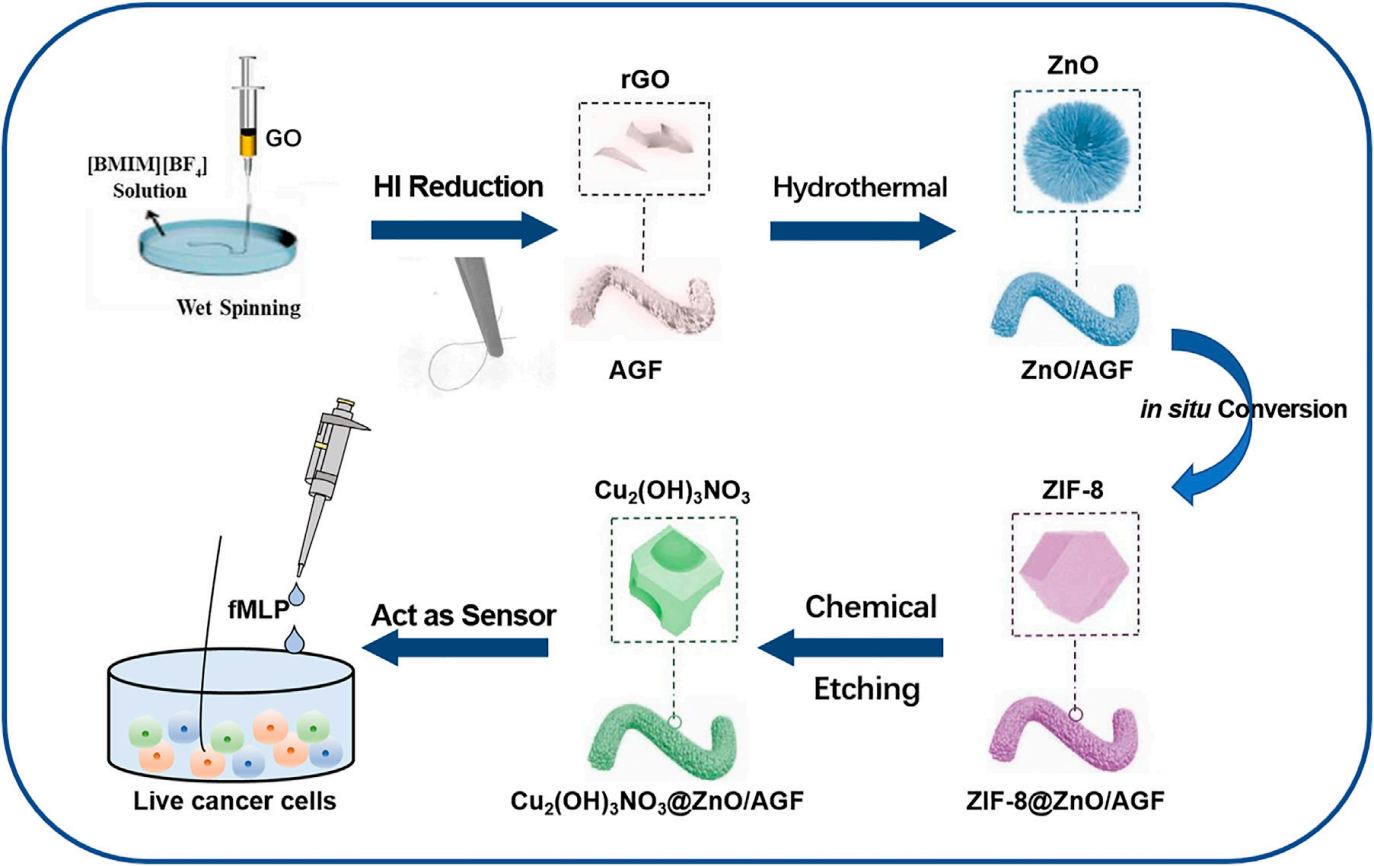
Fabrication procedure of a rime-like Cu_2_(OH)_3_NO_3_@ZnO/AGF microelectrode for live cancer cell detection.

In this work, we have developed an activated graphene fiber (AGF) modified with MOF-mediated rime-like hierarchical nanozyme as flexible and biocompatible microelectrode for *in situ* electrochemical detection of H_2_O_2_ in human colon cells. As far as we know, this has never been reported previously. As shown in Figure 1, the freestanding and flexible AGF is synthesized by wet-spinning using graphene oxide as the structural units and ionic liquid (IL) as coagulation bath to introduce heteroatoms (Cheng et al., 2015). The introduction of heteroatoms into the graphene materials not only possesses superior charge mobility to deliver high electrochemical activity, but also provides abundant active sites for the accessibility of substrate molecules and anchoring extrinsic functional species. To improve its catalytic activity, a metal-organic framework (MOF)-mediated rime-like hierarchical nanozyme [i.e.*,* Cu_2_(OH)_3_NO_3_@ZnO] is assembled on heteroatom-doped GF (Cui et al., 2016). Specifically, ZnO nanorods are grown on AGF *via* a typical solvothermal method. ZIF-8 is then *in situ* grown on ZnO nanorods by the impregnation method to obtain ZIF-8@ZnO/AGF. The Cu_2_(OH)_2_(NO_3_) is bonded to ZnO/AGF by chemical etching of ZIF-8, named, Cu_2_(OH)_3_NO_3_@ZnO/AGF. Benefitting from the synergistic contributions of dual nanozymatic activity of rime-like hierarchical Cu_2_(OH)_3_NO_3_@ZnO as well as unique structural and electrical properties, the resultant rime-like hierarchical Cu_2_(OH)_3_NO_3_@ZnO-decorated heteroatom-doped GF [Cu_2_(OH)_3_NO_3_@ZnO/AGF] microelectrode demonstrated significantly improved sensing performances to H_2_O_2_ detection, with a low detection limit (LOD) of 1 µM (S/N = 3) and a sensitivity of 272 μA cm^−2^ mM^−1^. These characteristics combined with its favorable anti-interference ability, high reducibility, and long-term stability, as well as good biocompatibility, enable a Cu_2_(OH)_3_NO_3_@ZnO/AGF microelectrode-based electrochemical biosensor to be used for real-time tracking of H_2_O_2_ released from different live human colon cells, which can act as a sensitive probe to distinguish colon cancer cells and normal colon epithelial cells. Thus, our strategy for the development of high-performance electrochemical biosensor coupled with recent advance in GF microelectrode and nanozyme opens up a new avenue for facile and efficient detection of possible disease-related clinical specimens, which is of great significance for the early diagnosis of various diseases in clinic practice.

## Experimental Section

### Chemicals and Materials

Sulfuric acid (H_2_SO_4_, 98%), hydrochloric acid (HCl, 37%), potassium nitrate (KNO_3_), potassium permanganate (KMnO_4_), hydrogen peroxide (H_2_O_2_, 30%), ethanol, zinc nitrate hexahydrate (Zn(NO_3_)_2_·6H_2_O), zinc acetate dihydrate (Zn(CH_3_COO)_2_)·2H_2_O, hexamethylenetetramine (C_6_H_12_N_4_), and copper nitrate trihydrate (Cu(NO_3_)_2_·3H_2_O) were purchased from the Sinopharm Chemical Reagent Co., Ltd. 1-Butyl-3-methylimidazolium tetrafluoroborate ([BMIM][BF_4_]), hydroiodic acid (HI, 48%), and worm-like expanded graphite powder (50 mesh number) were purchased from Shanghai Aladdin Biochemical Technology Co. Ltd. Guanine (G), adenine (A), cytosine (C), uracil (U), ascorbic acid (AA), D(+)-glucose (Glu), uric acid (UA), and dopamine (DA) were purchased from the Macklin Co. Ltd. N-formylmethionyl-leucyl-phenyl-alanine (fMLP, ≥99.5%) was obtained from Sigma-Aldrich (United States). All chemicals and reagents are of analytical grade and used without any further purification.

### Instruments

SEM images were acquired on Nova NanoSEM 450 (10 kV). TEM images were examined by Tecnai G2-F20 (200 kV). X-ray photoelectron spectroscopy (XPS) measurements were carried out on a Perkin-Elmer model PHI 5600 XPS system; all peaks were standardized to C 1s line at 284.6 eV correction. Powder x-ray diffraction (PXRD) was performed on a Rigaku D/max-IIIA diffractometer (Cu Kα, λ = 1.54056 Å) at room temperature. Electrochemical experiments were conducted on a CHI 760E electrochemical workstation (Shanghai CH Instruments Co., China).

### Synthesis of GF

Graphene oxide (GO) was first synthesized from worm-like expanded graphite powder based on a modified Hummer’s method (Xu et al., 2016). The as-obtained GO suspension was centrifuged twice at 14,000 rpm for 30 min to obtain concentrated GO suspension with a mass concentration of 20 mg ml^−1^. For wet spinning of GO fiber, the concentrated GO suspension was transferred into a 5-ml syringe connected by polyetheretherketone tube with a glass capillary (inner diameter of 500 μm) at the other end (Supplementary Figure S1). It was pumped into [BMIM][BF_4_] IL coagulation bath solution at a rate of 450 μl min^−1^. Subsequently, GO fibers were immersed into 30 ml of HI solution and kept at 80°C for 12 h to be reduced. Finally, they were washed successively with water and methanol to remove the HI and the iodine and dried at 60°C for 12 h.

### Synthesis of Cu_2_(OH)_3_NO_3_@ZnO/AGF Microelectrode

Reduced GF was placed in 30 ml of 30% H_2_O_2_ solutions at 70°C for 30 min and then washed with ultrapure water to obtain AGF. For the synthesis of ZnO nanorod-modified graphene fibers (ZnO/AGF), Zn(NO_3_)_2_ 6H_2_O (59.50 mg) and hexamethylenetetramine (28.04 mg) were dissolved in 40 ml of deionized water and stirred for 10 min. AGF was placed in a PTFE autoclave containing the above solution for 12 h at 90°C. Then, the obtained fibers were placed into a high-temperature tube furnace, and heated to 400°C at a heating rate of 5°C min^−1^ for 40 min to obtain ZnO/AGF. The as-synthesized ZnO/AGF was incubated into 2-methylimidazole solution (1.5 M, 30 ml) for 24 h for the *in situ* growth of ZIF-8 (ZIF-8@ZnO/AGF). Then, the ZIF-8@ZnO/AGF was soaked in Cu(NO_3_)_2_ solution (0.5 M) for 5–10 min to obtain Cu_2_(OH)_3_NO_3_@ZnO/AGF.

### Cell Culture

Human colon cells SW-48, NCM-460, and HCT-116 were obtained from the American Type Culture Collection (ATCC, Manassas, VA, United States). Cells were seeded in a 6-well plate for 24 h, and grown in Dulbecco’s modified eagle medium containing 10% fetal bovine serum supplemented with 100 units ml^−1^ penicillin and 100 mg ml^−1^ streptomycin in an incubator at 37°C with 5% CO_2_. The incubation solution was removed after growing to 90% confluence, then washed with PBS for three times to collect cells. The number of cells was counted by a hemocytometer. For real-time measurements of H_2_O_2_ in live cells, a three-electrode system was located on the plate containing 3 ml of PBS with the cell density of 5 × 10^6^ cells ml^−1^, and the amperometric responses of different live cells were recorded.

### Cell Detection

Nikon Ti-U microscope was used to record the color changes of fluorescent dichlorofluorescein. The microscope was equipped with a CSU-X1 spinning-disk confocal unit (Yokogawa) and an EM-charge-coupled device camera (iXon+; Andor). The chronoamperometry (*i-t*) measurement was performed at an applied potential of −0.8 V vs. Ag/AgCl. After the baseline was stable, 10 μl of fMLP (1 mg ml^−1^) was injected into wells (1 ml) of different types of colon cell, and then the responding currents were recorded to monitor the concentration of H_2_O_2_.

## Results and Discussion

### Morphological and Structural Characterization

**FIGURE 2:**
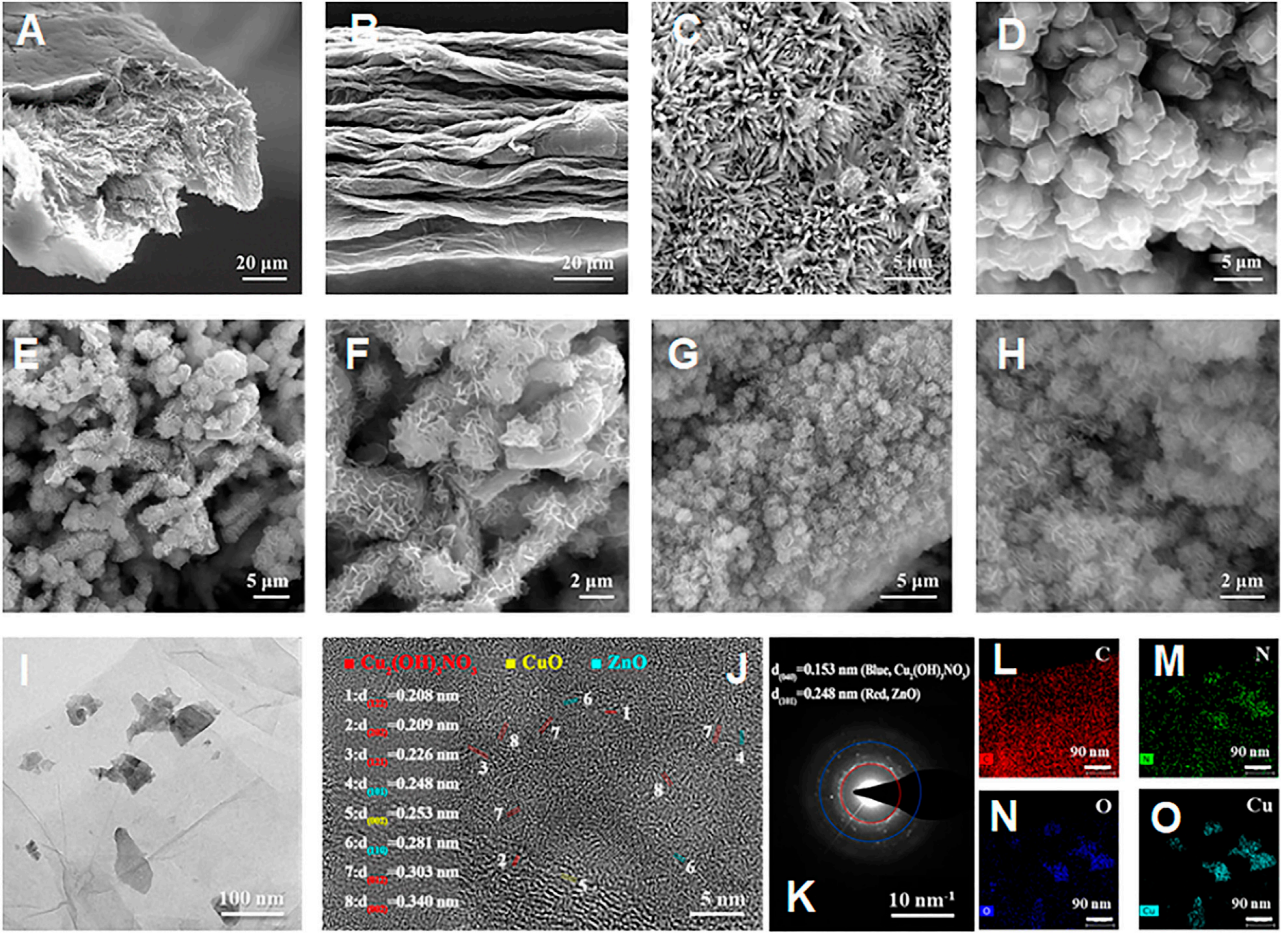
SEM images of **(A)** cross-sectional view and **(B)** front view of the AGF; SEM images of **(C)** ZnO/AGF and **(D)** ZIF-8@ZnO/AGF; SEM images of **(E–H)** Cu_2_(OH)_3_NO_3_@ZnO/AGF at different magnifications; TEM image of **(I)** Cu_2_(OH)_3_NO_3_@ZnO/AGF; **(J)** HRTEM image and corresponding **(K)** SAED pattern of Cu_2_(OH)_3_NO_3_@ZnO/AGF; **(L–O)** EDS mappings of C, Cu, N, O, and Zn elements in Cu_2_(OH)_3_NO_3_@ZnO/AGF.

For the preparation of hierarchical nanohybrid microelectrode, the flexible GF has been used as the freestanding electrode substrate. As shown in Figures 2A,B, it can be observed that GF consists of stacked graphene nanosheets neatly aligned along the axis to form a layered structure with a uniform diameter of ∼50 μm and wrinkled structure (Figure 2B). The wrinkled structure endows the GF with a typical porous microstructure, which provides a larger specific surface area. For the subsequent growth of nanohybrids, the pristine GF is oxidized by chemical methods to introduce the desired functional groups on its surface to enhance its hydrophilicity. The ZnO is synthesized by a hydrothermal method with a rod-like morphology (Figure 2C), endowing ZnO/AGF with a larger surface area than bare AGF, which is conducive to easier loading of other active materials in subsequent derivatization steps. After the coordination of 2-methylimidazole with zinc ions, the ZnO nanorods are *in situ* converted to ZIF-8 with rhombic dodecahedron morphology. The average particle size of ZIF-8 is about 3 μm with smooth and uniform surface (Figure 2D). Then, ZIF-8 is used as a mediator to facilitate the growth of Cu_2_(OH)_3_NO_3_ nanozyme on ZnO nanorods. As shown in Figures 2E–H, ZIF-8 with rhombic dodecahedron is etched into irregular particles, while the inner part of the particles still has a rod-shaped ZnO skeleton. The morphology of Cu_2_(OH)_3_NO_3_@ZnO/AGF resembles rime in natural landscape (Supplementary Figure S2). The nanostructure of Cu_2_(OH)_3_NO_3_@ZnO/AGF has also been investigated by transmission electron microscopy (TEM). Figure 2I reveals that Cu_2_(OH)_3_NO_3_@ZnO/AGF possesses ultrathin nanosheet morphology. The ultrathin nanosheet morphology further endows the microelectrode with more exposed active sites and high electrocatalytic activity towards H_2_O_2_. High-resolution TEM (HRTEM) image shows that the lattice fringe with a crystal spacing of 0.248 and 0.153 nm matches well with the crystal plane ZnO (101) and Cu_2_(OH)_3_NO_3_ (040), respectively (Figure 2J). The corresponding selected area electron diffraction (SAED) pattern presents some clear bright spots, further confirming the composition of Cu_2_(OH)_3_NO_3_@ZnO/AGF (Figure 2K). The HAADF-STEM and the corresponding energy-dispersive spectroscopy (EDS) mappings demonstrate the distribution of C, N, O, Cu, and Zn in Cu_2_(OH)_3_NO_3_@ZnO/AGF (Figures 2L–O), further confirming the successful synthesis of Cu_2_(OH)_3_NO_3_@ZnO/AGF.

**FIGURE 3:**
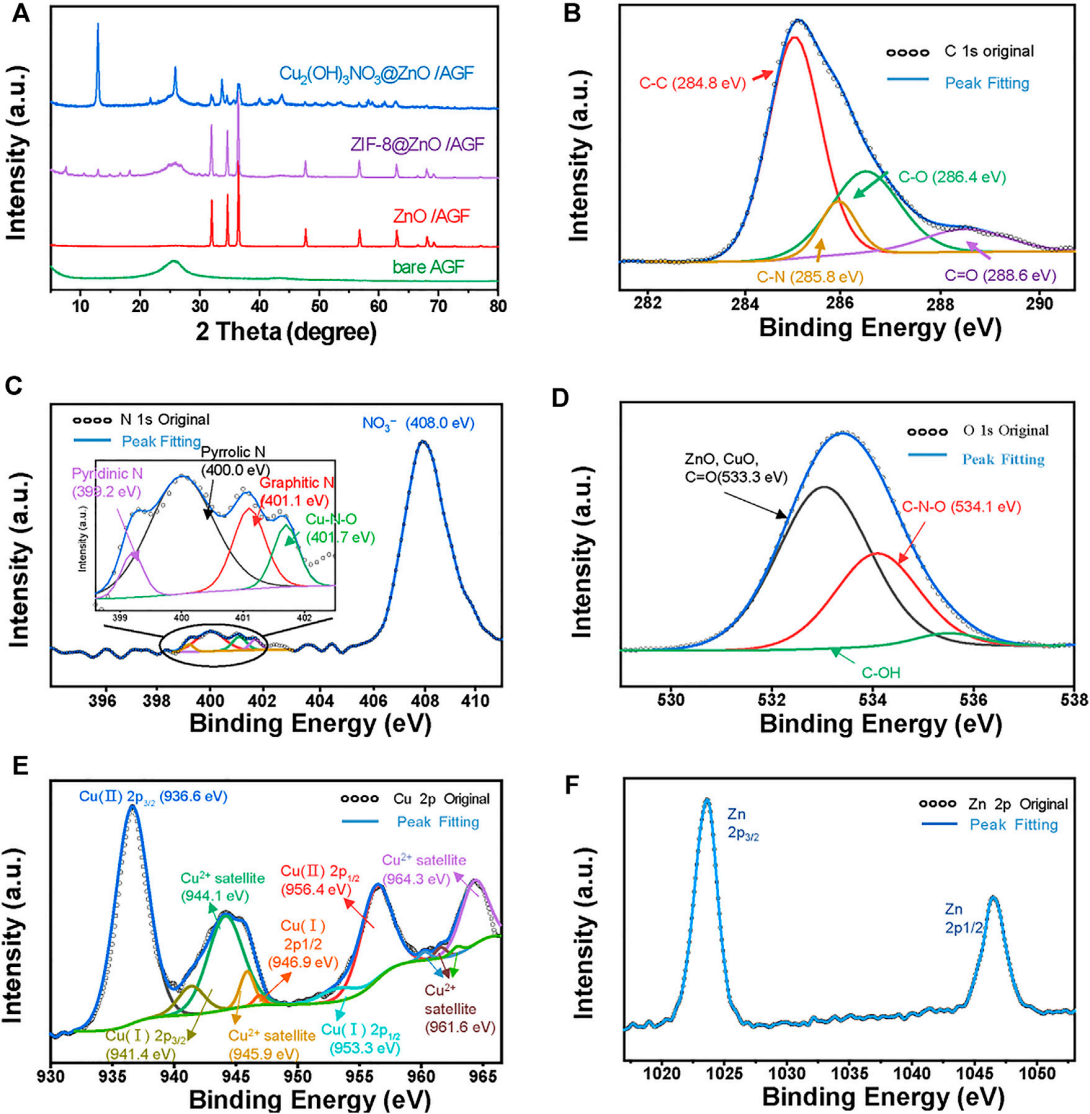
**(A)** PXRD patterns of AGF, ZnO/AGF, ZIF-8@ZnO/AGF, and Cu_2_(OH)_3_NO_3_@ZnO/AGF. High-resolution XPS spectra of **(B)** C 1s, **(C)** N 1s, **(D)** O 1s, **(E)** Cu 2p, and **(F)** Zn 2p regions in Cu_2_(OH)_3_NO_3_@ZnO/AGF.

The composition of hierarchical nanohybrids has been investigated by powder x-ray diffraction (PXRD) analysis. Figure 3A shows that the PXRD patterns of AGF exhibit a broad peak at 26.4°, corresponding to the crystallographic planes of C (002). After decorating with ZnO, four distinct characteristic diffraction peaks are observed at 31.7°, 34.4°, 36.2°, and 47.4°, corresponding to the (100), (002), (101), and (102) planes of ZnO (PDF#36-1451) (Anila et al., 2021). A series of peaks with weak intensity appeared at 5°–20° in the ZIF-8@ZnO/AGF pattern, which can be attributed to the diffraction peaks of ZIF-8. The PXRD pattern of Cu_2_(OH)_3_NO_3_@ZnO/AGF exhibits new peaks at 12.8°, 25.7°, 33.6°, 35.3°, 36.4°, 39.8°, 43.5°, and 49.2°, corresponding to the crystallographic planes of (001), (002), (−201), (−121), (121), (−202), (122), and (−131) of Cu_2_(OH)_3_NO_3_ (PDF#15-0014) (Supplementary Figure S3), which are well consistent with the HRTEM characterization (Munyemana et al., 2020). Thermogravimetric analysis (TGA) under N_2_ atmosphere provides stability information of Cu_2_(OH)_3_NO_3_@ZnO/AGF, which shows a two-step weight loss (Supplementary Figure S4). TG curve shows a sharp weight loss beyond 225°C associated with the decomposition of Cu_2_(OH)_3_NO_3_, followed by a long plateau until 700°C. The mass loss at 700°C corresponds to the reduction of ZnO by carbon at high temperature to generate carbon dioxide or carbon monoxide. These results demonstrate the successful synthesis of hierarchical nanohybrids on AGF.

The surface chemical states of different elements in Cu_2_(OH)_3_NO_3_@ZnO/AGF have further been verified by x-ray photoelectron spectroscopy (XPS) analysis. The survey spectrum exhibits five predominant peaks at 285, 400, 533, 930, and 1,030 eV, assigned to C 1s, N 1s, O 1s, Cu 2p, and Zn 2p, respectively (Supplementary Figure S5). The surface atomic ratio of C:N:O:Cu:Zn is 29.87:8.42:44.46:10.59:6.65. The corresponding fitting curves of these elements are depicted in Figures 3B–F. The C 1s spectra can be deconvoluted into four components corresponding to different functional groups. The peaks at 284.8, 285.8, 286.4, and 288.6 eV are designated as C-C, C-N, C-O, and C=O, respectively. The high-resolution spectrum of N 1s shows a strong intensity of peak at 408.0 eV and a smaller intensity of peak in the range of 399–402 eV. The strong intensity of peak is assigned to NO_3_^−^ and the smaller intensity of peak region is deconvoluted into four peaks at 399.2, 400.0, 401.1, and 401.7 eV, which are further assigned to pyridinic N, pyrrolic N, graphitic N, and N-oxide/Cu, respectively (Zhuang et al., 2014). The O 1s spectrum of Cu_2_(OH)_3_NO_3_@ZnO/AGF can be deconvoluted into three spin-orbit doublets, the fitting peaks at a binding energy of 533.3, 534.1, and 535.0 eV corresponding well with C=O, C-N-O, and C-O respectively. The Cu 2p core level XPS spectrum exhibits the Cu^2+^ 2p_3/2_ peak at 936.6 eV and the Cu^2+^ 2p_1/2_ peaks at 956.4 eV, with an energy gap of 19.8 eV, indicating that Cu exhibits only one oxidation valence of +2. The Zn 2p core level XPS spectrum exhibits the Zn 2p_3/2_ peak at 1,023 eV and the Zn 2p_1/2_ peaks at 1,047 eV, with an energy gap of 24 eV. All XPS results indicated that the successful synthesis of hierarchical dual nanozyme Cu_2_(OH)_3_NO_3_@ZnO on AGF.

### Electrochemical Characterization of Microelectrodes

**FIGURE 4:**
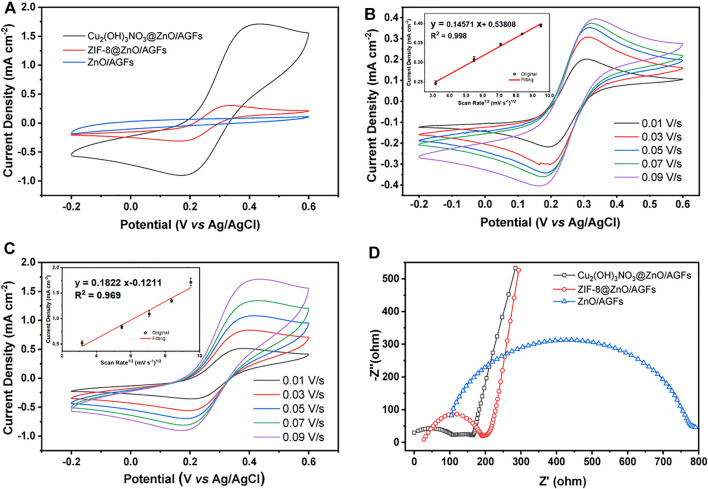
**(A)** CV curves of different microelectrodes in 0.1 M KCl solution containing 5.0 mM [Fe(CN)_6_]^3−/4−^. Scan rate: 0.09 V s^−1^. CV measurements of **(B)** ZIF-8@ZnO/AGF and **(C)** Cu_2_(OH)_3_NO_3_@ZnO/AGF are performed with [Fe(CN)_6_]^3−/4−^ as the redox probe at different scan rates from 0.01 to 0.09 V s^−1^. **(D)** Nyquist plots of ZnO/AGF, ZIF-8@ZnO/AGF, and Cu_2_(OH)_3_NO_3_@ZnO/AGF in 5 mM [Fe(CN)_6_]^3−/4−^ solution. Frequency range: 0.01–10^6^ Hz.

The electrochemical properties of the stepwise fabrication process of nanohybrid microelectrodes, such as current responses (*I*_pa_) and peak-to-peak separation (Δ*E*_p_), are studied by CV in 0.1 M KCl solution containing 5.0 mM [Fe(CN)_6_]^3-/4-^. The voltammetric curves for ZIF-8@ZnO/AGF and Cu_2_(OH)_3_NO_3_@ZnO/AGF exhibit a pair of well-defined quasi-reversible peaks (Figure 4A), which signify the oxidation/reduction process by outer-sphere electrode reaction of the redox couple. However, ZnO/AGF does not show a pair of well-defined quasi-reversible peaks, which is due to the insulator characteristics of ZnO. The current response is sequentially improved by modification with ZIF-8@ZnO and Cu_2_(OH)_3_NO_3_@ZnO nanohybrids, respectively. The oxidation and reduction peak currents of Cu_2_(OH)_3_NO_3_@ZnO/AGF are approximately 24 times higher than that of ZnO/AGF. However, the peak separation is increased after modification, which should be attributed to the non-porous structure of Cu_2_(OH)_3_NO_3_. Furthermore, CVs of the stepwise fabrication process of nanohybrid microelectrodes in contact with 0.1 M KCl solution containing 5.0 mM [Fe(CN)_6_]^3–/4–^ at different scan rates from 0.01 to 0.09 V s^−1^ are recorded. Figures 4B,C and Supplementary Figure S6 show that the current response increases with the increase in scan rate. As seen in Figures 4B,C inset and Supplementary Figure S6 inset, plots of anodic (*I*_pa_) peak currents against square root of scan rate show the great linearity, suggesting that the reaction mechanism is diffusion-controlled. Based on the Randles-Sevick equation, the electroactive surface area of the modified electrodes is calculated to be 0.0091, 0.0351, and 0.2995 cm^2^ for ZnO/AGF, ZIF-8@ZnO/AGF, and Cu_2_(OH)_3_NO_3_@ZnO/AGF, respectively. Compared to ZnO/AGF, the peak current densities of the modified electrodes are drastically increased in the order of ZnO/GF < ZIF-8@ZnO/GF < Cu_2_(OH)_3_NO_3_@ZnO/AGF, indicating that decorating Cu_2_(OH)_3_NO_3_@ZnO on the surface of AGF-based microelectrodes remarkably improves the electroactive surface area and increases the rate of electron transfer between active sites and the redox species in solution.

To investigate the charge transport mechanisms of various fabricated microelectrodes, electrochemical impedance spectroscopy (EIS) measurements are conducted using [Fe(CN)_6_]^3–/4–^ as redox probes, where the Nyquist plots can be fitted by an equivalent circuit (Supplementary Figure S7). Typically, the semicircular part of the Nyquist plot in the EIS coincides with the electron transfer-dependent process, where the diameter represents the interfacial charge transfer resistance (*R*_ct_), and the low frequency of the linear part is attributed to the diffusion-dependent process (Ciucci 2019). As shown in Figure 4D, there are larger *R*_ct_ values of 504.4 Ω for ZnO/AGF. The *R*_ct_ decreases to 141.4 Ω after ZIF-8 *in situ* grown on ZnO/AGF, which should be attributed to the porosity of ZIF-8 facilitating charge transport to GF by ions in solution. When the Cu_2_(OH)_3_NO_3_ is bonded to ZnO/AGF by chemical etching of ZIF-8, the *R*_ct_ value is further decreased to 52.8 Ω, which is due to the positive charge of [Cu_2_(OH)_3_]^+^ (the electrostatic attraction between positively charged`` [Cu_2_(OH)_3_]^+^ and negatively charged [Fe(CN)_6_]^3–/4–^ facilitates the charge transfer of [Fe(CN)_6_]^3–/4–^ on the electrode surface). All the above EIS results indicate that the electrochemical sensing platforms for H_2_O_2_ are fabricated successfully as expected.

### Electrochemical Sensing Performance of the Cu_2_(OH)_3_NO_3_@ZnO/AGF Microelectrode

**FIGURE 5:**
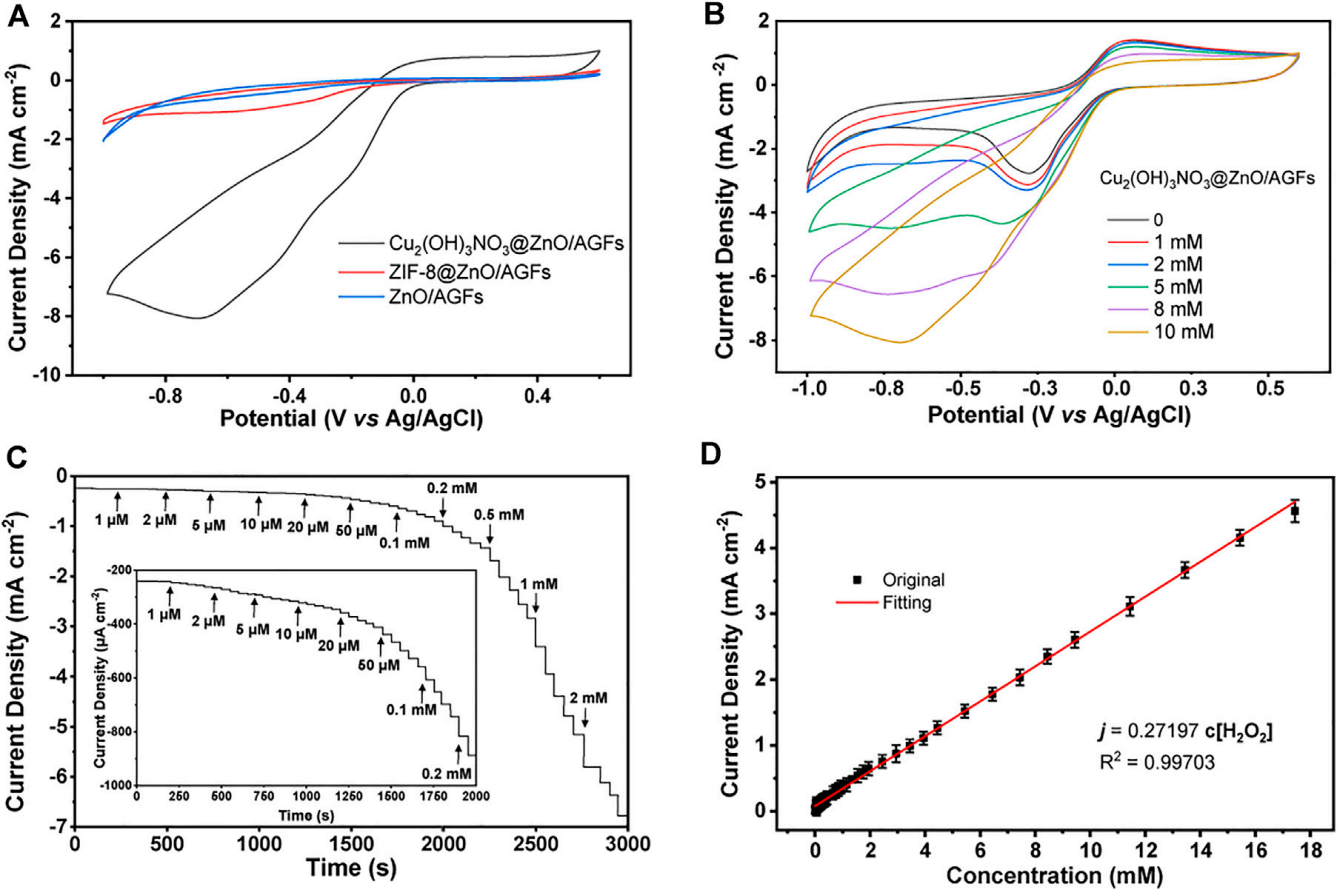
**(A)** CV curves of ZnO/AGF, ZIF-8@ZnO/AGF, and Cu_2_(OH)_3_NO_3_@ZnO/AGF in 0.1 M PBS solution (pH 7.4) containing 5 mM H_2_O_2_. **(B)** CV curves of Cu_2_(OH)_3_NO_3_@ZnO/AGF in 0.1 M PBS solution (pH 7.4) containing different concentrations of H_2_O_2_. Scan rate: 50 mV s^−1^. **(C)** Amperometric current responses of Cu_2_(OH)_3_NO_3_@ZnO/AGF upon stepwise addition of different concentrations gradients of H_2_O_2_ in stirred 0.1 M PBS solution (pH 7.4). Inset: Amperometric current responses of Cu_2_(OH)_3_NO_3_@ZnO/AGF at low concentrations. Applied potential: −0.8 V. **(D)** Linear relationship between the corresponding current and H_2_O_2_ concentration.

Considering the unique hierarchical structure of Cu_2_(OH)_3_NO_3_@ZnO/AGF, the electrocatalytic performance of different microelectrodes towards H_2_O_2_ has been investigated by CV measurements in 0.1 M PBS (pH 7.4) solution containing 5 mM H_2_O_2_. As shown in Figure 5A, the CV curve of Cu_2_(OH)_3_NO_3_@ZnO/AGF shows a clear reduction peak at −0.8 V, and the peak current density is much higher than that of ZnO/AGF and ZIF-8@ZnO/AGF. With the addition of H_2_O_2_, the reduction current increases linearly, indicating that Cu_2_(OH)_3_NO_3_@ZnO/AGF possesses high electrocatalytic activity towards H_2_O_2_ (Figure 5B). However, CV curves of ZIF-8@ZnO/AGF and ZnO/AGF show relatively low activity towards H_2_O_2_ (Supplementary Figure S8). This result can be explained in the following two aspects: (I) From a structure point of view, after *in situ* conversion of ZnO nanorods to ZIF-8, ZIF-8@ZnO/AGF possesses a larger surface area and adsorption capacity due to the unique porous structure of the metal-organic framework, benefiting the catalytic activity of ZIF-8@ZnO/AGF towards H_2_O_2_. In addition, a redox couple of Cu^2+^/Cu^+^ is introduced by the metal salt impregnation method to etch ZIF-8 into rime-like Cu_2_(OH)_3_NO_3_, which greatly improves the catalytic activity of Cu_2_(OH)_3_NO_3_@ZnO/AGF. (II) From an electrochemical perspective, the charge transfer from Cu_2_(OH)_3_NO_3_@ZnO to AGF microelectrode leads to a slight change in the electronic structure of Cu_2_(OH)_3_NO_3_, which further improves their electrocatalytic performance. In terms of the catalytic mechanisms, the exposure of Cu^2+^/Cu^+^ sites can act as a facilitating center to promote H_2_O_2_ activation. For the reaction process on Cu_2_(OH)_3_NO_3_, Cu_2_ (OH)_3_NO_3_ is partially reduced *in situ* into Cu_2_(OH)NO_3_ during electrochemical treatment (Yi et al., 2020). Then, the generated Cu_2_(OH)NO_3_ are oxidized again to Cu_2_(OH)_3_NO_3_ in the presence of H_2_O_2_. The mechanism process of Cu_2_(OH)_3_NO_3_@ZnO/AGF to catalyze the decomposition of H_2_O_2_ can be presented as follows (Ling et al., 2020):

$${\text{Cu}}_{2}{\left(\text{OH}\right)}_{3}{\text{NO}}_{3}+2e^{-}\to \text{Cu}\left(\text{OH}\right){\text{NO}}_{3}+2{\text{OH}}^{-}$$ (1)

$${\text{Cu}}_{2}\left(\text{OH}\right){\text{NO}}_{3}+{\text{H}}_{2}{\text{O}}_{2}\to {\text{Cu}}_{2}{\left(\text{OH}\right)}_{3}{\text{NO}}_{3}$$ (2)

Furthermore, the amperometric measurements are applied to evaluate the sensitivity of Cu_2_(OH)_3_NO_3_@ZnO/AGF upon stepwise addition of different concentrations of H_2_O_2_ in 0.1 M PBS solution with stirring (pH 7.4), where an optimal applied potential of −0.8 V is selected for testing. As shown in Figure 5C, a fast response signal can be observed with the addition from 1 μM to 2 mM H_2_O_2_, reaching 95% of the steady-state current within 3 s. The significant drop of the reduction current can be attributed to the rapid diffusion and activation of H_2_O_2_ on the active sites of the rime-like Cu_2_(OH)_3_NO_3_ with high specific surface, which could facilitate the fast electron transfer kinetics. Figure 5D shows that the proposed electrochemical sensor displays a wide linear range of the reduction current versus H_2_O_2_ concentration in the range of 1 μM–17.4 mM with a sensitivity value of 272 μA cm^−2^ mM^−1^. The calculated LOD is as low as 1 µM (S/N = 3). These results are better than or comparable with those of most H_2_O_2_ sensors reported in the recently published literature (Supplementary Table S1).

**FIGURE 6:**
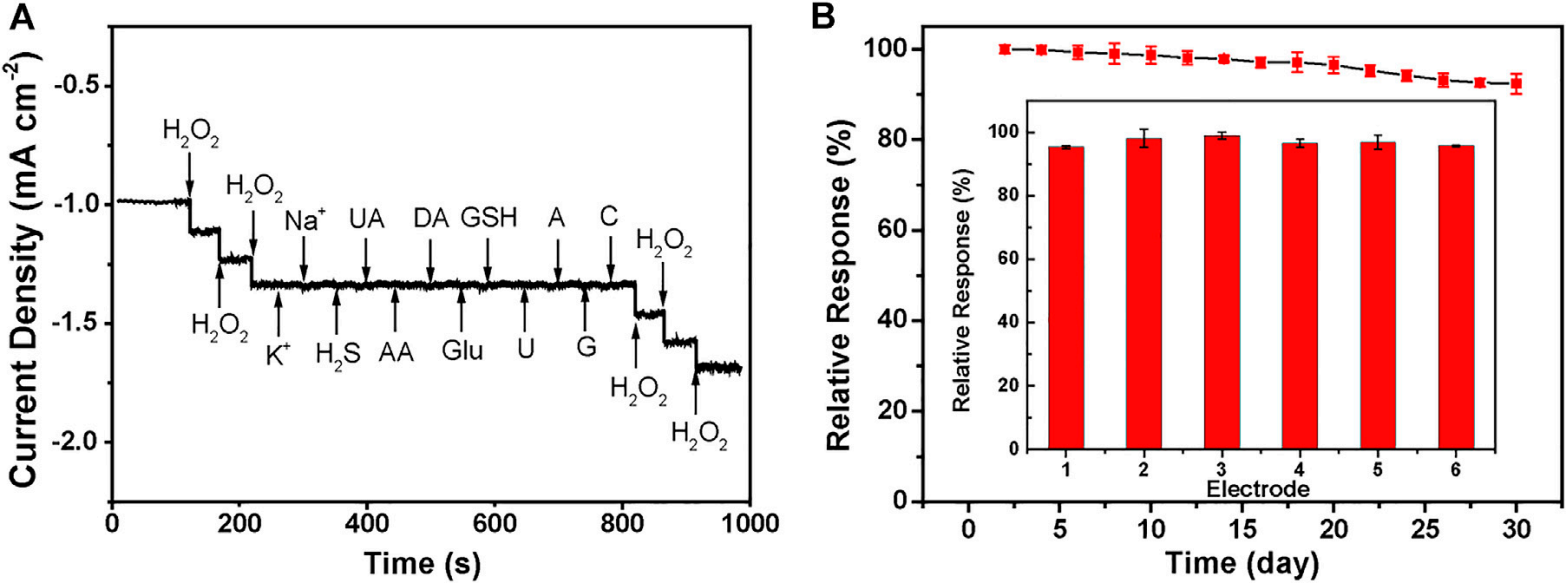
**(A)** Amperometric current response curves of Cu_2_(OH)_3_NO_3_@ZnO/AGF to 0.2 mM H_2_O_2_, KCl, NaCl, H_2_S, UA, AA, Glu, DA, GSH, DA, U, A, C, and G. Applied potential: −0.8 V. **(B)** Current responses of six different Cu_2_(OH)_3_NO_3_@ZnO/AGF to 0.2 mM H_2_O_2_. Inset: Current responses of Cu_2_(OH)_3_NO_3_@ZnO/AGF to 0.2 mM H_2_O_2_ for 4 weeks.

Anti-interference is another essential parameter of H_2_O_2_ sensors. In this work, the injection of 1.0 mM interfering species, i.e., K^+^, Na^+^, H_2_S, UA, AA, Glu, DA, GSH, DA, U, A, C, and G almost does not cause distinctive amperometric current density changes compared with 0.2 mM H_2_O_2_ under the negative applied potential of −0.8 V, demonstrating the preferential selectivity of Cu_2_(OH)_3_NO_3_@ZnO/AGF towards the detection of H_2_O_2_ (Figure 6A). The reproducibility of the proposed flexible electrode is also evaluated by successive monitoring of 0.2 mM H_2_O_2_ with six modified electrodes prepared under the same procedure. The relative standard derivation value of their amperometric current responses is measured to be only 5% (Figure 6B inset). In addition, the current response maintains 90% of its initial current value after 4 weeks by periodically recording the current response to 0.2 mM H_2_O_2_, indicative of its good long-term stability (Figure 6B).

### Monitoring of H_2_O_2_ in Live Cells

**FIGURE 7:**
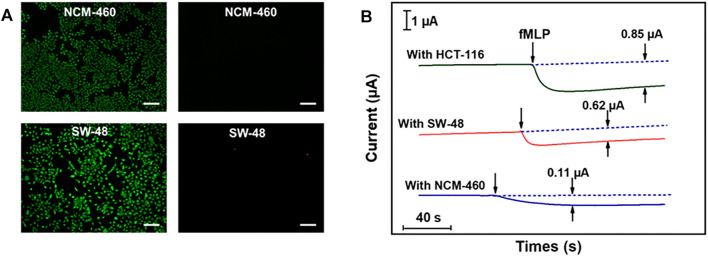
**(A)** Live and dead body fluorescence images of SW-48 and NCM-460 cells, detected by Calcein-AM/PI, with Calcein-AM staining surviving cells green and PI staining dead cells red. Scale bar, 100 μm. **(B)** Amperometric current responses of Cu_2_(OH)_3_NO_3_@ZnO/AGF to the addition of 10 μM fMLP in test solution containing 5.0 × 10^6^ NCM-460, SW-48, and HCT-116 cells.

Colon cancer is a clinically common human gastrointestinal malignancy with the third highest incidence worldwide and the fifth highest mortality rate among all malignancies in China, and the only way to improve survival rate as well as to escape from these deadly attacks is through early diagnosis (Wang et al., 2021). Therefore, the practical application of the proposed Cu_2_(OH)_3_NO_3_@ZnO/AGF microelectrode has been explored for tracking intracellular H_2_O_2_ fluctuations in different human colon cells, i.e., colon cancer cells (SW-48 and HCT-116), and epithelial cell lines from normal colon (NCM-460). Cytotoxicity tests show that Cu_2_(OH)_3_NO_3_@ZnO/AGF microelectrode does not induce cell death (apoptosis and necrosis) in SW-48 and NCM-460 cells (Figure 7A). fMLP is a kind of chemotactic peptide, used as a stimulator to initiate the release of H_2_O_2_ from living cells. The amperometric curves will exhibit an obvious reduced current density after injecting an amount of fMLP in the test cell containing different human body cells. Afterwards, the released H_2_O_2_ is quantified by electrochemical measurement using Cu_2_(OH)_3_NO_3_@ZnO/AGF as probe. Figure 7B shows the amperometric current responses to the addition of 10 μM fMLP in test solution containing 5.0 × 10^6^ cells. The amperometric current values increase by 0.11, 0.62, and 0.85 μA for NCM-460, SW-48, and HCT-116, respectively. These results indicate that human colon cancer cells produce more extracellular H_2_O_2_ than normal cells, which is consistent with the titration measurement observation (Supplementary Table S2). Thus, the Cu_2_(OH)_3_NO_3_@ZnO/AGF-based electrochemical sensor can be applied to effectively differentiate normal and tumor cells for cancer cell detection.

## Conclusion

In summary, we have developed a new type of high-performance flexible microelectrode based on MOF-mediated hierarchical nanohybrid-modified AGF, and used it in the electrochemical sensing system. Our results show that due to the synergistic effects of dual nanozymatic activity of rime-like hierarchical Cu_2_(OH)_3_NO_3_@ZnO as well as their unique structural and electrical properties, excellent sensitivity, selectivity, long-term stability, reproducibility, and practicality, the Cu_2_(OH)_3_NO_3_@ZnO/AGF microelectrode shows remarkable electrochemical sensing performance towards H_2_O_2_. The resultant electrochemical sensing platforms can be used for real-time tracking of H_2_O_2_ released from different kinds of live colon cells, which provides an effective strategy to distinguish cancer cells from the normal one for clinical diagnosis. An extension to more complex systems can be foreseen, upon conducting process/device engineering with this system. We envision that this work will open up a new pathway for the design of an assisting technology for cancer diagnosis and treatments in the future to hold great potential in the development of advanced implantable and wearable smartsensor for clinical practices.

## Data Availability

The original contributions presented in the study are included in the article/Supplementary Material, further inquiries can be directed to the corresponding author.
