# Supplementary material for: Functionalized Graphene Fiber Modified With MOF-Derived Rime-Like Hierarchical Nanozyme for Electrochemical Biosensing of H2O2 in Cancer Cells
Source: Front Chem. 2022 Mar 22;10:873187. doi: 10.3389/fchem.2022.873187 (PMC8980740; doi:10.3389/fchem.2022.873187)
Supplement: Supplementary file 1 [file DataSheet1.docx]

Supplementary Material

Functionalized Graphene Fiber Modified with MOF-Derived Rime-Like Hierarchical Nanozyme for Electrochemical Biosensing of H_2_O_2_ in Cancer Cells

Wei Huang^2^, Yun Xu^2^, Yimin Sun^1*^

^1^ Hubei Key Laboratory of Plasma Chemistry and Advanced Materials, School of Materials Science and Engineering, Wuhan Institute of Technology, Wuhan, China

^2^ Key Laboratory of Material Chemistry for Energy Conversion and Storage, Ministry of Education, School of Chemistry and Chemical Engineering, Huazhong University of Science & Technology, Wuhan, China

*** Correspondence:**Yimin Sun
ymsun@wit.edu.cn

## Supplementary Figures


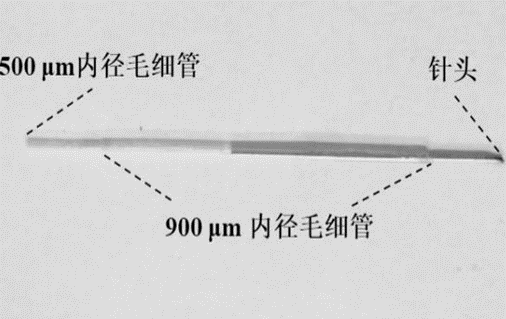


**Supplementary Figure S1.** Photo of 5 mL syringe connected by polyetheretherketone (PEEK) tube, while the other end fitted with a glass capillary (inner diameter of 500 μm)


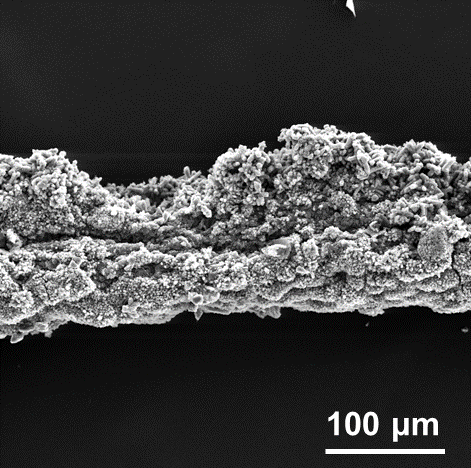


**Supplementary Figure S2.** SEM image of a rime-like Cu_2_(OH)_3_NO_3_@ZnO/AGF microelectrode.

**Supplementary Figure S3.** PXRD spectra of Cu_2_(OH)_3_NO_3_@ZnO/AGF and PDF pattern of Cu_2_(OH)_3_NO_3_.

**Supplementary Figure S4.** Thermogravimetry of Cu_2_(OH)_3_NO_3_@ZnO/AGF upon heating at a rate of 5 °C/min in N_2_.

**Supplementary Figure S5.** XPS energy survey spectrum of Cu_2_(OH)_3_NO_3_@ZnO/AGF.

**Supplementary Figure S6.** CV measurements of ZnO/AGFs were performed with [Fe(CN)_6_]^3−/4−^ as the redox probe at different scan rates from 0.01 to 0.09 V s^-1^.


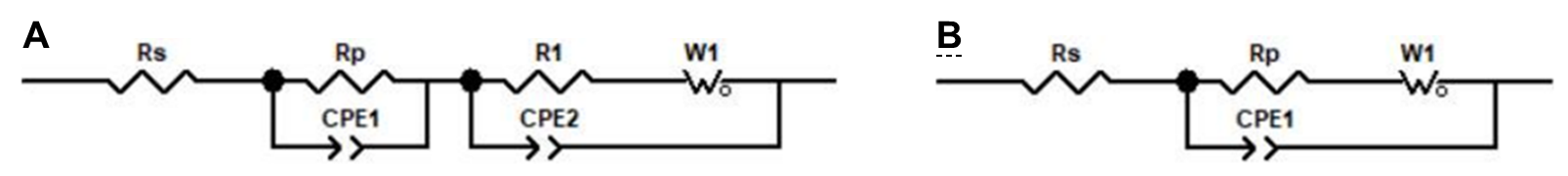


**Supplementary Figure S7.** The equivalent circuit used to fit the experimental EIS data of (A) Cu_2_(OH)_3_NO_3_@ZnO/AGF and (B) ZnO/AGFs and ZIF-8@ZnO/AGFs.


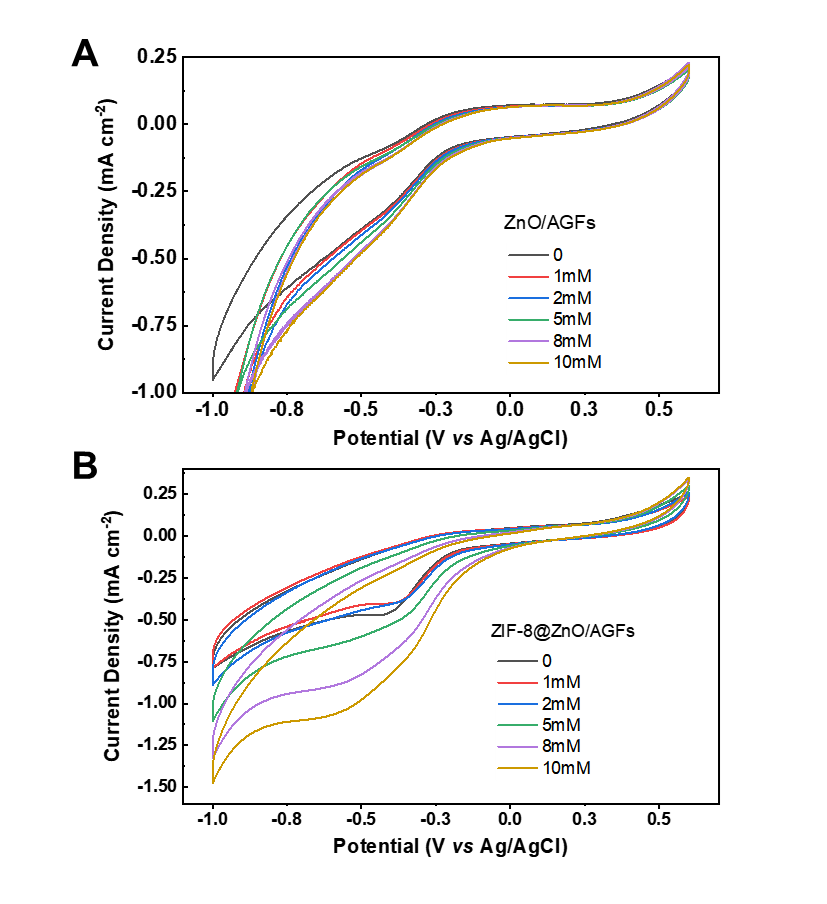


**Supplementary Figure S8.** CV curves of (A) ZnO/AGFs and (B) ZIF-8@ZnO/AGFs in 0.1 M PBS solution (pH 7.4) containing different concentrations of H_2_O_2_. Scan rate: 50 mV s^−1^.

## Supplementary Table

**Supplementary Table S1**

| Sample | Linear range | Sensitivity  (μA mM^-1^ cm^-2^) | LOD | Reference | |
| --- | --- | --- | --- | --- | --- |
| Cu(OH)_3_NO_3_@ZnO/AGF | 4 μM-18 mM | 272 | 1 μM | | This work |
| vG/NRs-F | 2-15 mM | 39.6 | 320 μM | | Hang et al. (2019) |
| Co_3_N NW/TM | 2 μM-28 mM | 139.9 | 1 μM | | Xie et al. (2018) |
| C/V_2_O_3_ NSs | 5.0-1500 μM | 204.64 | 1.7 μM | | Chen et al. (2021) |
| NiO-NSs/CF | 0.20-3.75 mM | 23.30 | 0.013 μM | | Liu et al. (2021) |
| Fe-SASC/NW | 0.5-0.5mM | - | 0.046 μM | | Ding et al. (2021) |
| γ-Fe_2_O_3_/Fe_3_O_4_ | 0.2 μM-8 mM | - | 0.05 μM | | Molodtsova et al. (2021) |
| GN@FeOOH | 0.25 μM-1.2 mM | 265.7 | 0.08 μM | | Chen et al. (2020) |
| Co@MOF-808 | 10-450 μM | 382.27 | 1.3 μM | | Chang et al. (2020) |
| CC/Co@C-CNTs | 0.40 μM-7.2 mM | 388 | 0.27 μM | | Long et al. (2020) |
| [Mo-oxo]n | 50 nM-15 mM | 2200 | 0.23 μM | | Liu et al. (2020) |
| MnO_2_-NWs@Au-NPs/GF | 10 μM-9.51 mM | 32.9 | 1.9 μM | | Zhao et al. (2020) |
| CNTs | 5 μM-23 mM | 104.9 | 0.138 μM | | Niamlaem et al. (2020) |
| FePc-CP NSs | 0.1-1000 μM | 97 | 0.017 μM | | Liu et al. (2019) |
| NiCo_2_S_4_/rGO | 0.025-11.25 mM | 118.5 | 0.19 μM | | Wang et al. (2019) |

**Supplementary Table S2**

To compare the amount of H_2_O_2_ released from human colon cells obtained from amperometric measurements, the filtrate was measured to cross-verify the amount of H_2_O_2_ released from different human colon cells. First, a 2 % starch indicator aqueous solution (2 mL) was added to the combined filtrate. The solution was then titrated by adding KI aqueous solution (0.005 M) dropwise until the solution color turned from transparent to blue.

|  | KI aqueous solution / mL | | |
| --- | --- | --- | --- |
| NCM-460 | 5.3 | 4.7 | 5.6 |
| SW-48 | 6.7 | 7.2 | 7.5 |
| HCT-116 | 8.2 | 8.5 | 8.8 |

**Supplementary References**

Chen, S., Wang, C., and Lu, X. (2021) Fabrication of Two-Dimensional Carbon/V_2_O_3_ Composite Nanosheets and Their Application for Electrochemical Sensing. *Compos. Commun.* 27, 100842. doi: [10.1016/j.coco.2021.100842](https://doi.org/10.1016/j.coco.2021.100842)

Chen, X., Gao, J., Zhao, G., and Wu, C. (2020). In Situ Growth of FeOOH Nanoparticles on Physically-Exfoliated Graphene Nanosheets as High Performance H_2_O_2_ Electrochemical Sensor. *Sens. Actuators, B* 313, 128038. doi: [10.1016/j.snb.2020.128038](https://doi.org/10.1016/j.snb.2020.128038)

Chang, Y-S., Li, J-H., Chen, Y-C., Ho, H., Song, Y-D. and Kung, C-W. (2020) Electrodeposition of Pore-Confined Cobalt in Metal-Organic Framework Thin Films toward Electrochemical H_2_O_2_ Detection. *Electrochim. Acta* 347, 136276. doi: [10.1016/j.electacta.2020.136276](https://doi.org/10.1016/j.electacta.2020.136276)

Ding, S., Lyu, Z., Fang, L., Li, T., Zhu, W., Li, S., et al. (2021) Single-Atomic Site Catalyst with Heme Enzymes-Like Active Sites for Electrochemical Sensing of Hydrogen Peroxide. *Small* 17, 2100664. doi: [10.1002/smll.202100664](https://doi.org/10.1002/smll.202100664)

Hang, T., Xiao, S., Yang, C., Li, X., Guo, C., He, G., et al. (2019). Hierarchical Graphene/Nanorods-Based H_2_O_2_ Electrochemical Sensor with Self-Cleaning and Anti-Biofouling Properties. *Sens. Actuators, B* 289, 15-23. doi: [10.1016/j.snb.2019.03.038](https://doi.org/10.1016/j.snb.2019.03.038)

Liu, M., An, M., Xu, J., Liu, T., Wang, L., Liu, Y., et al. (2021) Three-Dimensional Carbon Foam Supported NiO Nanosheets as non-Enzymatic Electrochemical H_2_O_2_ Sensors. *Appl. Surf. Sci*. 542, 148699. doi: [10.1016/j.apsusc.2020.148699](https://doi.org/10.1016/j.apsusc.2020.148699)

Liu, R., Luo, Y., Zheng, Y., Zhang, G., and Streb, C. (2020) Polyoxometalate-Like Sub-Nanometer Molybdenum(VI)-oxo Clusters for Sensitive, Selective and Stable H_2_O_2_ Sensing. *Chem. Commun.* 56, 9465-9468. doi: [10.1039/D0CC03758C](https://doi.org/10.1039/D0CC03758C)

Liu, W., Pan, H., Liu, C., Su, C., Liu, W., Wang, K., et al. (2019) Ultrathin Phthalocyanine-Conjugated Polymer Nanosheet-Based Electrochemical Platform for Accurately Detecting H_2_O_2_ in Real Time. *ACS Appl. Mater. Interfaces* 11, 11466-11473. doi: [10.1021/acsami.8b22686](https://doi.org/10.1021/acsami.8b22686)

Long, L., Liu, H., Liu, X., Chen, L., Wang, S., Liu, C., et al. (2020) Co Embedded N-Doped Hierarchical Carbon Arrays with Boosting Electrocatalytic Activity for In Situ Electrochemical Detection of H_2_O_2_. *Sens. Actuators, B* 318, 128242. doi: [10.1016/j.snb.2020.128242](https://doi.org/10.1016/j.snb.2020.128242)

Molodtsova, T., Gorshenkov, M., Saliev, A., Vanyushin, V., Goncharov, I., and Smirnova, N. (2021). One-Step Synthesis of γ-Fe_2_O_3_/Fe_3_O_4_ Nanocomposite for Sensitive Electrochemical Detection of Hydrogen Peroxide. *Electrochim. Acta* 370, 137723. doi: [10.1016/j.electacta.2021.137723](https://doi.org/10.1016/j.electacta.2021.137723)

Niamlaem, M., Boonyuen, C., Sangthong, W., Limtrakul, J., Zigah, D., Kuhn, A., et al. (2020) Highly Defective Carbon Nanotubes for Sensitive, Low-Cost and Environmentally Friendly Electrochemical H_2_O_2_ Sensors: Insight into Carbon Supports. *Carbon* 170, 154-164. doi: [10.1016/j.carbon.2020.07.081](https://doi.org/10.1016/j.carbon.2020.07.081)

Wang, M., Ma, J., Guan, X., Peng, W., Fan, X., Zhang, G., et al. (2019) A Novel H_2_O_2_ Electrochemical Sensor Based on NiCo_2_S_4_ Functionalized Reduced Graphene Oxide. *J. Alloys Compd.* 784, 827-833. doi: [10.1016/j.jallcom.2019.01.043](https://doi.org/10.1016/j.jallcom.2019.01.043)

Xie, F., Cao, X., Qu, F., Asiri, A. M., and Sun, X. (2018). Cobalt Nitride Nanowire Array as an Efficient Electrochemical Sensor for Glucose and H_2_O_2_ Detection. *Sens. Actuators, B* 255, 1254-1261. doi: [10.1016/j.snb.2017.08.098](https://doi.org/10.1016/j.snb.2017.08.098)

Zhao, A., She, J., Manoj, D., Wang, T., Sun, Y., Zhang, Y., et al. (2020) Functionalized Graphene Fiber Modified by Dual Nanoenzyme: Towards High-Performance Flexible Nanohybrid Microelectrode for Electrochemical Sensing in Live Cancer Cells. *Sens. Actuators, B* 310, 127861. doi: [10.1016/j.snb.2020.127861](https://doi.org/10.1016/j.snb.2020.127861)
